# Supplementary material for: Information content of contact-pattern representations and predictability of epidemic outbreaks
Source: Sci Rep. 2015 Sep 25;5:14462. doi: 10.1038/srep14462 (PMC4585889; doi:10.1038/srep14462)
Supplement: Supplementary Information [file srep14462-s1.pdf]

Supplementary material Fig. S1

Information content of contact-pattern representations and predictability of epidemic outbreaks

Petter Holme

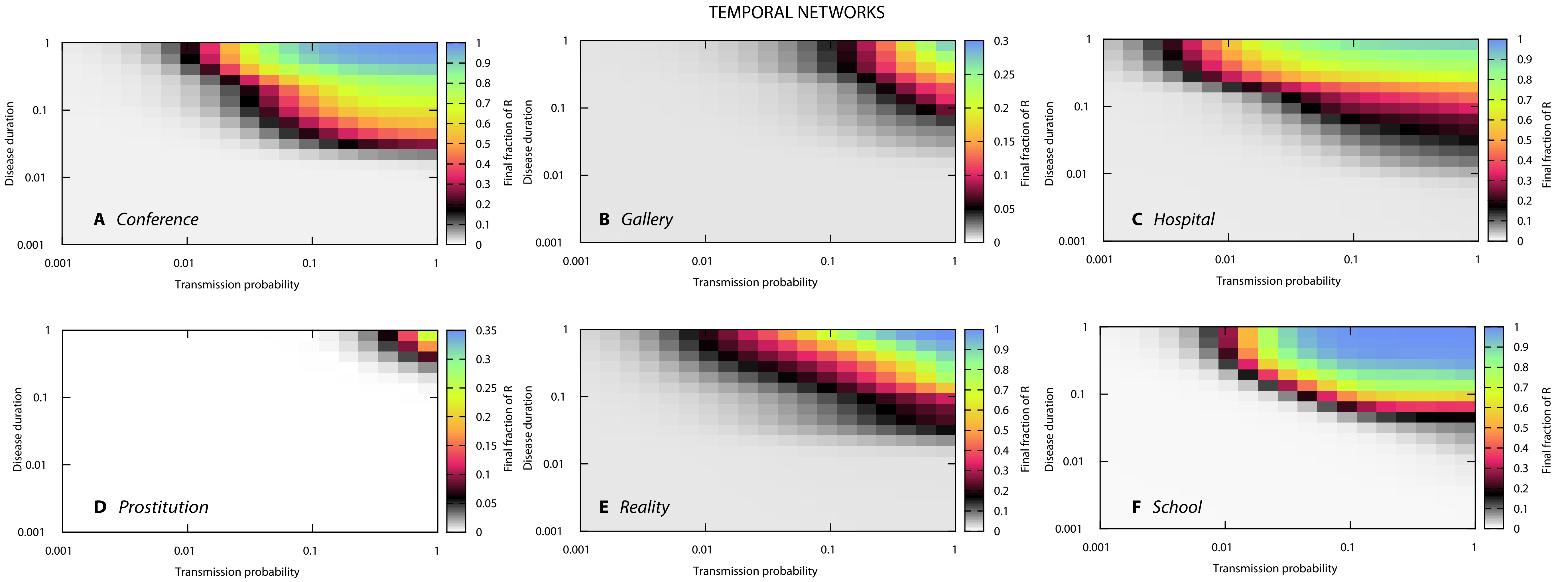

TEMPORAL NETWORKS
A Conference
B Gallery
C Hospital
D Prostitution
E Reality
F School
Disease duration
Transmission probability
Final fraction of R
0.001
0.01
0.1
1
0
0.05
0.1
0.15
0.2
0.25
0.3
0.35
0.4
0.5
0.6
0.7
0.8
0.9

STATIC NETWORKS

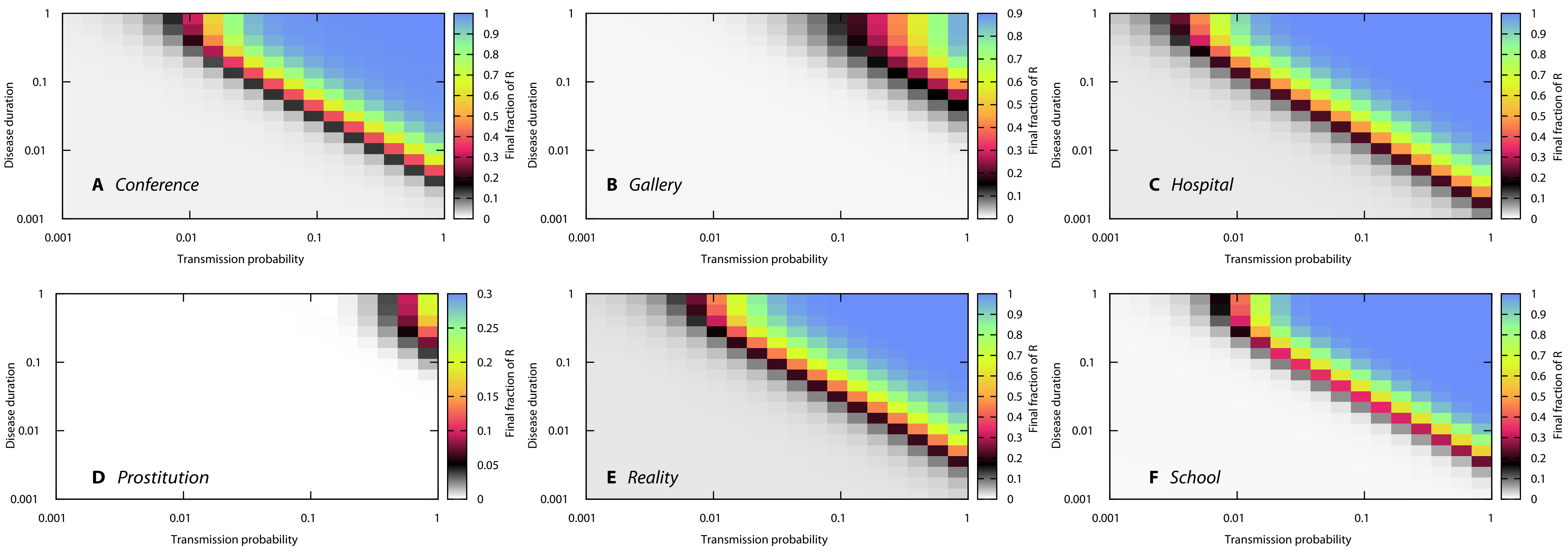

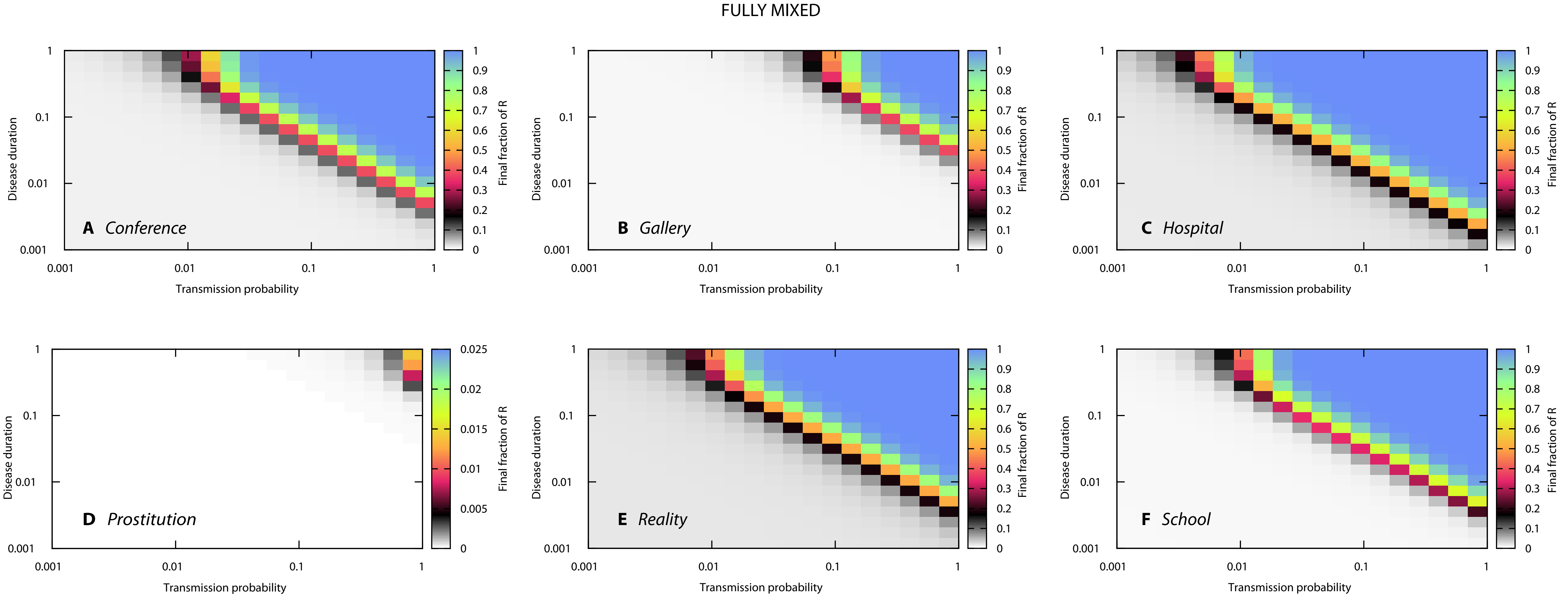
FULLY MIXED
A Conference
B Gallery
C Hospital
D Prostitution
E Reality
F School
Disease duration
Transmission probability
Final fraction of R
0.001
0.01
0.1
1
0
0.1
0.2
0.3
0.4
0.5
0.6
0.7
0.8
0.9
0.005
0.015
0.02
0.025

Supplementary material Fig. S2

Information content of contact-pattern representations and predictability of epidemic outbreaks

Petter Holme

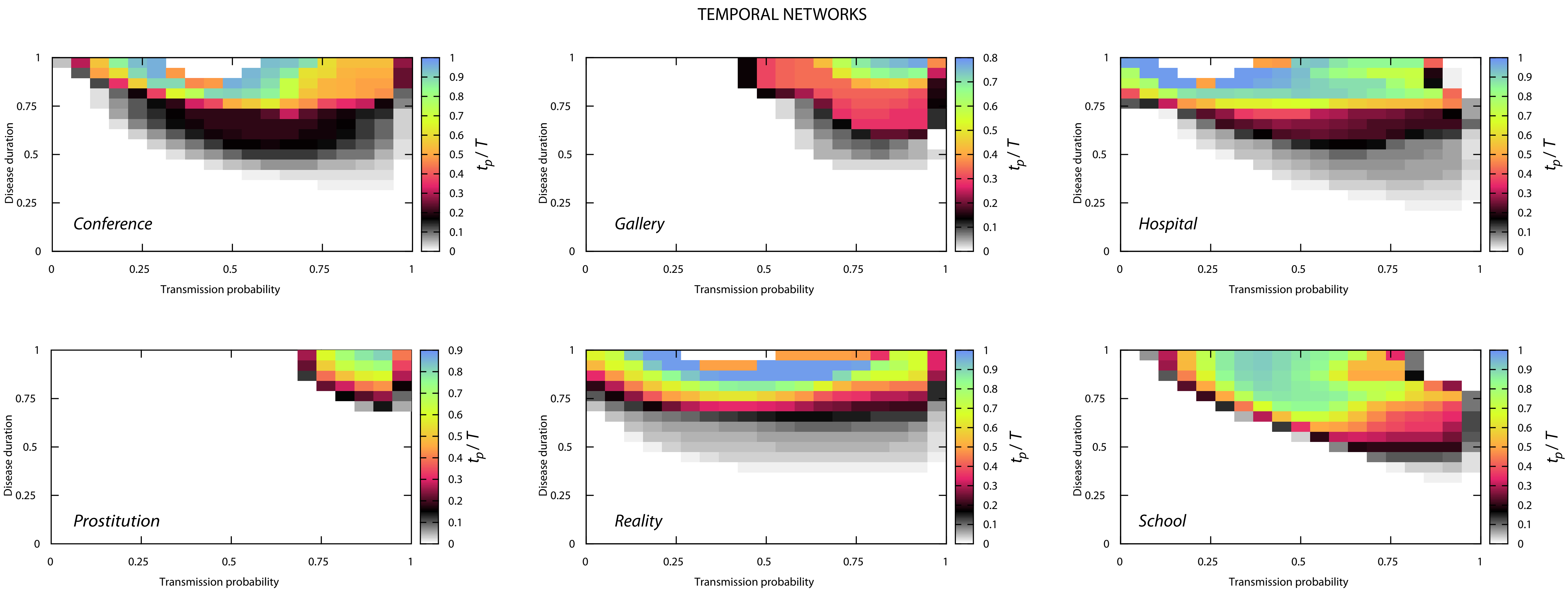
TEMPORAL NETWORKS
Conference
Gallery
Hospital
Prostitution
Reality
School
Disease duration
Transmission probability
t_p / T
0
0.25
0.5
0.75
1
0.1
0.2
0.3
0.4
0.5
0.6
0.7
0.8
0.9

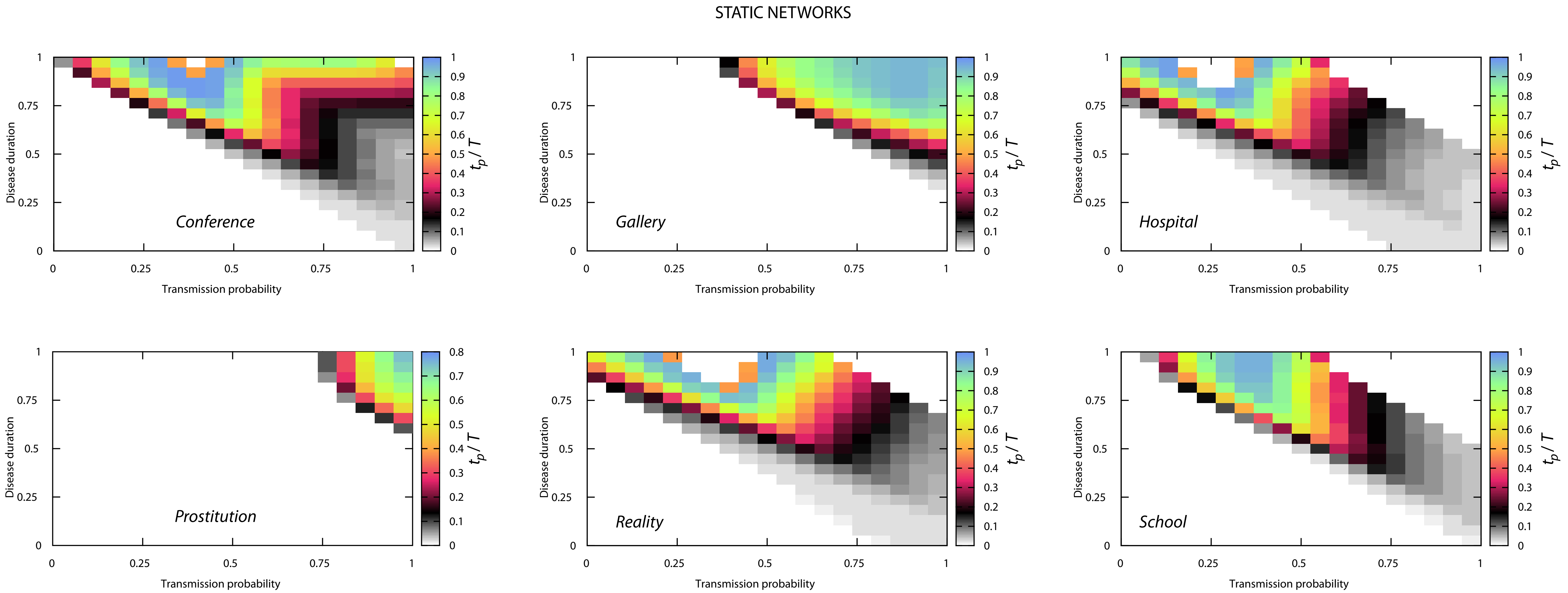
STATIC NETWORKS
Conference
Gallery
Hospital
Prostitution
Reality
School
Disease duration
Transmission probability
t_p / T
0
0.25
0.5
0.75
1

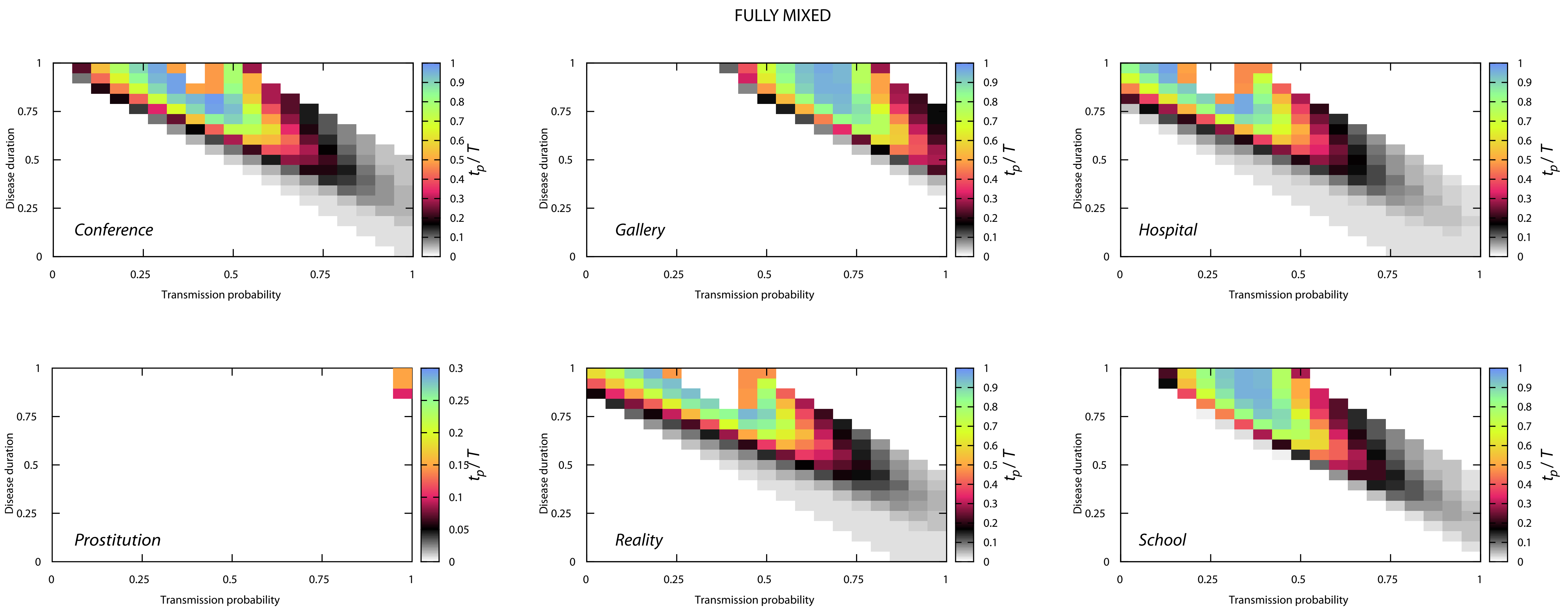
FULLY MIXED
Conference
Gallery
Hospital
Prostitution
Reality
School
Disease duration
Transmission probability
t_p / T
